# Risky Sexual Behaviors and Associated Factors among Jiga High School and Preparatory School Students, Amhara Region, Ethiopia

**DOI:** 10.1155/2016/4315729

**Published:** 2016-06-15

**Authors:** Getachew Mullu Kassa, Genet Degu, Meseret Yitayew, Worku Misganaw, Mikiyas Muche, Tiguaded Demelash, Meless Mesele, Melat Ayehu

**Affiliations:** ^1^Midwifery Department, Medicine and Health Sciences College, Debre Markos University, P.O. Box 269, Debre Markos, Ethiopia; ^2^Nursing Department, Medicine and Health Sciences College, Debre Markos University, P.O. Box 269, Debre Markos, Ethiopia

## Abstract

*Background*. Young people constitute a large number of population worldwide, and majority of this population group lives in developing countries. They are at high risk of engaging in risky sexual behaviors. These risk sexual behaviors predispose youths to several sexual and reproductive health problems like STIs, HIV, unwanted pregnancy, and abortion. So, this study was conducted to assess the magnitude of risky sexual behaviors and associated factors among Jiga high school and preparatory school students, northwest Ethiopia.* Methodology*. Institutional based cross-sectional study design was conducted among Jiga town high school and preparatory school students. A total of 311 students were included in the study. Systematic random sampling method was used to select study participants. Data was entered using EpiData version 3.1 and it was exported to SPSS version 22 for further analysis. Descriptive analysis and bivariate and multivariate analysis were also calculated to determine factors associated with risky sexual behavior.* Result*. Forty-eight (16%) of respondents reported that they had sexual intercourse. From those who start sex, 44 (14.7%) were involved in risky sexual behavior which could predispose them to sexual and reproductive health problems. More than half, 27 (56.3%), of respondents first sexual intercourse was before their eighteenth birthday. The mean age and SD of fist sexual initiation were 17.2 years old and 1.35 years, respectively. Factors associated with risky sexual behavior include respondents between the ages of 20 and 23 (AOR: 5, 95%, CI: 1.59–15.98), drinking alcohol (AOR: 2.48, 95% CI: 1.13–5.41), and having poor knowledge towards HIV/AIDS (AOR: 4.53, 95%, CI: 2.06–9.94).* Conclusion*. A large number of in-school youths are involved in risky sexual behaviors like early sexual initiation, having multiple sexual partners, inconsistence use of condom, and having sex with high risk partner (CSWs). Age of respondents, alcohol drinking, and poor knowledge towards HIV/AIDS were factors associated with risky sexual behavior. School and community based programs in reducing substance abuse among youths and increasing their knowledge towards HIV/AIDS are important.

## 1. Introduction

More than half of the world population constitutes of young people's less than 25 years old, and majority of these populations live in developing country [1]. Young peoples are at high risk of practicing high risk sexual behaviors, because of the risk taking behavior during this age group [2, 3].

In developing countries, the rate of risky sexual behaviors including unprotected sexual intercourse and early sexual initiation is increasing [4]. Studies have showed that more than 50% of new sexually transmitted diseases (STDs) every year are due to young peoples aged 5 to 24 [5]. Young peoples are engaged in high risk behaviors like smoking cigarettes, drinking alcohol, use of drugs, and gender based violence. These behaviors in turn lead them to engage in sexual risk behaviors [6]. In Ethiopia and many other developing countries, the epidemic of HIV is high among young peoples which is mainly due to the risky sexual behavior [7]. The United Nation's Program on HIV/AIDS (UNAIDS) report on the Global AIDS Epidemic showed that sub-Saharan Africa accounts for 60% of all peoples living with HIV/AIDS [8].

Risk sexual behavior is a behavior related to sexuality which increases the susceptibility of an individual to problems related to sexuality and reproductive health like sexually transmitted disease (STIs), human immune deficiency virus (HIV), unwanted and unplanned pregnancy, abortion, and psychological distress [9–16]. Risk behavior includes having more than one sexual partner, early sexual initiation, inconsistent use of condom, and having sex with commercial sex workers [9, 12–17]. Additionally, the use of substances during sex may engage young peoples in risky sexual behaviors since it affects their judgment [18]. Alcohol use is cited by several studies as it is one of the common factors which increases the risk of HIV acquisition [19].

Such risky sexual behaviors increase the risk of youths to acquire HIV [20]. Addressing the issue of sexual and nonsexual risk behaviors among young peoples is important in reducing the risk of HIV and other STI diseases [6]. So, this study was conducted to assess the magnitude of risky sexual behaviors and associated factors among Jiga high school and preparatory school students, northwest Ethiopia.

## 2. Materials and Methods

### 2.1. Study Area and Period

The study was conducted in Jiga woreda, West Gojjam, Amhara, Ethiopia. The woreda is 373 km far from Addis Ababa, the capital city of Ethiopia. The woreda has one high school and one preparatory school. According to 2007 national census conducted by the central statistical agency of Ethiopia (CSA), the woreda has a total population of 28,402 from this 13,917 are male and 14,485 are females. The study was conducted from April to May 2014.

### 2.2. Study Design and Study Population

School based cross-sectional study design was conducted among Jiga high school (grades 9-10) and preparatory school (grades 11-12) students. Study participants were randomly selected from the study population. Regular grade 9 to 12 students were included in to the study, while students who were absent during the data collection period and those who were unable to respond to the questionnaire due to serious illness were excluded from the study.

### 2.3. Sample Size Determination and Sampling Procedure

Sample size for the study was determined by using single population proportion formula by considering 30% prevalence of risky sexual behavior, sexually active students, from a study conducted in Oromia region, Bale zone [21], 95% confidence interval (CI), 5% margin of error, and 10% nonresponse rate. In addition, since the total number of students in the schools was 2,216, less than 10,000 correction formula was used, which gives a final sample size of 311. Respondents were selected by using a systematic random sampling method, after proportionally allocating the total number of participants for each grade (grades 9–12).

### 2.4. Study Variables and Operational Definition

The dependent variable of this study was risky sexual behavior, and the independent variables were sociodemographic characteristics (like age, sex, educational and occupational level, and income), knowledge towards HIV/AIDS, and substance abuse (like alcohol drinking, smoking, chat chewing, and use of drugs like shish/hashish).

Risky sexual behavior is defined as sexually active school students who have at least one of the following: multiple sexual partners, having more than one sexual partner before the data collection period; sexual initiation before the age 18; inconsistent use of condom (incorrect use of condom or failure to use condom at least once during sexual intercourse); and sexual intercourse with commercial sex workers. Respondents who answered/score more than the mean of correct answers for HIV/AIDS related questions were categorized to have good knowledge, while those who responded below the mean of correct answer were classified as having poor knowledge towards HIV/AIDS.

### 2.5. Data Collection Procedures

Structured self-administered questionnaire, adapted and modified from other study [21], was used to collect data. The questionnaire was first prepared in English then translated to the local language, Amharic, by a person who have good knowledge of both languages. Data was collected by five nursing students from Debre Markos University (DMU).

### 2.6. Data Processing and Analysis

EpiData version 3.1 software was used to enter the collected data. Then, the data was exported to SPSS software version 22 for further analysis. Descriptive and inferential statics was used to summarize the collected data. Bivariate and multivariate analysis was used to identify factors associated with the dependent variable, at *p* value less than 0.05.

### 2.7. Ethical Consideration

Ethical clearance was obtained from DMU, College of Medicine and Health Science. Prior to the data collection, permission letter was obtained from different levels at Jiga town. Informed consent was obtained from the study participants, after providing the necessary information on the objective, significance, and confidentiality issues of the study.

## 3. Results

### 3.1. Sociodemographic Characteristics of Respondents

The current study was conducted to assess the risky sexual behaviors and associated factors among Jiga high school and preparatory school students. From a sample of 310, a total of 300 respondents completed the questionnaire which gives a response rate of 96.8%. Majority, 165 (55%), of respondents were females. Almost two-thirds (62.3%) of respondents were in the age group of greater than or equal to 18 years old. The mean and standard deviation (SD) for age of respondents were 17.75 years and 1.37 years, respectively. Most, 92%, of respondents were not married, and 97.7% were orthodox religion followers. One hundred eighty-eight (62.7%) of respondents currently live with their mothers and fathers, while 25 (8.3%) live alone (Table 1).

### 3.2. Sexual History of Respondents

Forty-eight (16%) of respondents reported that they had sexual intercourse prior to the data collection period. From those who start sex, 44 (14.7%) were involved in risky sexual behavior which could predispose them to sexual and reproductive health problems. More than half, 27 (56.3%), of respondents who start sex said that their first sexual intercourse was before their eighteenth birthday. The mean age and SD of first sexual initiation were 17.2 years old and 1.35 years old, respectively. The main reason given by respondents to stat sex was peer pressure followed by personal desire, each accounting for 35.4% and 27.1% of the reasons, respectively. Only 8 (16.7%) respondents who start sex used condom during the first sex, while 40 (83.3%) did not use condom at first sex. In addition, 3 respondents who start sex had sexual intercourse with commercial sex worker (CSW), and 14 (29.2%) had multiple sexual partner prior to the data collection period. Twenty-eight (58.3%) had sexual intercourse in the past twelve months (Table 2).

### 3.3. Knowledge of Respondents towards HIV/AIDS

Majority, 93.3%, of respondents were aware of HIV/AIDS, while 20 (6.7%) respondents reported that they have never heard of HIV/AIDS. Respondents were also asked to mention the modes of HIV transmission. The main mode of HIV transmission listed by respondents was sexual contact, followed by injection with contaminated needles, and mother to child transmission, each accounting for 264 (88%), 216 (72%), and 213 (71%), respectively. Eight (2.7%) and 4 (1.3%) of the respondents said that HIV can be transmitted by hand shaking and by air. Regarding the overall knowledge score, 192 (64%) respondents had good knowledge towards HIV/AIDS, while 108 (36%) had poor knowledge. In addition, only 19 (64.3%) respondents agreed with the idea that condoms prevent pregnancy, and 57 (19%) and 102 (34%) disagree and do not know that condoms prevent STIs if used properly (Table 3).

### 3.4. Substance Abuse by Respondents

One hundred thirty-two of the respondents said that they drank alcohol prior to the data collection period. In addition 14 (4.7%), 7 (2.3%), and 5 (1.7%) of the respondents also said that they chew chat, smoke cigarette, and use shisha/hashish, respectively (Table 4).

### 3.5. Factors Associated with Risky Sexual Behavior

Bivariate and multivariate analysis was computed using SPSS software to identify factors associated with risky sexual behavior. On bivariate analysis, variables which were found to have association with risky sexual behavior include respondents between the ages 20 and 23 (COR: 3.4, 95% CI: 1.51–7.56); educational level of grade 11 (COR: 0.27, 95% CI: 0.09–0.75); drinking alcohol (COR: 2.87, 95% CI: 1.47–5.62); ever chewed chat (COR: 6.73, 95% CI: 2.23–20.28); ever use of shish/hashish (COR: 9.29, 95% CI: 1.51–57.32); and having poor knowledge towards HIV/AIDS (OR: 3.85, 95% CI: 1.97–7.51).

Multivariate logistic regression was also done to identify the independent effect of the variables by controlling the confounding effect of other variables. Accordingly, three variables were found to have association with risky sexual behavior at *p* value less than 0.05. These include respondents between the ages of 20 and 23 (AOR: 5, 95% CI: 1.59–15.98); drinking alcohol (AOR: 2.48, 95% CI 1.13–5.41); and having poor knowledge towards HIV/AIDS (AOR: 4.53, 95% CI: 2.06–9.94) (Table 5).

## 4. Discussion

This study assessed the magnitude of risky sexual behavior and associated factors among Jiga high school and preparatory school students.

This study illustrated that 16% of respondents had sex prior to the data collection period. This finding is lower than a study conducted at Humera, northwest Ethiopia, 21.8% and a study conducted in western Ethiopia, 35.3% [13, 15]. Similar findings conducted in Jimma University showed that 26.9% of respondents started sexual intercourse [22]. This is higher than the current finding. The reason for such difference could be attributed to the difference in sociodemographic characteristics and study population. In addition, the mean age at first sexual intercourse in this study was 17.2 years. This finding is almost similar with study conducted in Jimma University with the mean age at first sexual intercourse being 17.7 years old [22].

More than half, 56.3%, of respondents start sexual intercourse before their eighteenth birthday. This finding is below the Ethiopian Demographic and Health Survey (EDHS) 2011 report, which showed that among women aged 25–49, 62 percent start sexual intercourse before age of 18 [23]. A study conducted in Nigerian University students showed a higher level of sexual initiation (76.8%) [24]. The possible reason for such difference when compared with the current finding could be due to the sociodemographic characteristics and cultural difference in relation to sexuality and reproductive health between the two countries [22].

From 48 respondents who had sex, only 8 (16.7%) used condom, and the rest 83.3% did not used condom at first sex. This kind of risky sexual behavior could predispose young peoples to several sexual and reproductive health problems [9]. Similar findings conducted in Tiss Abay showed that 88.9% of respondents who start sex did not use condom at first sex [9]. This is slightly higher than the current study finding. The possible explanation for the difference could be the difference in sample size and the study population; the study in Tiss Abay included only female youths, while the current study includes both male and female in-school youths. The United States national survey on youth risk behavior report also showed that 40% of teenagers engage in sexual activity without the use of condom [25].

The magnitude of risky sexual behavior in the current study area was found to be 14.7%. From this, 56.3% start sex before the age of 18, 83.3% did not use condom at first sex, 6.3% had sex with CSW, and 29.2% had multiple sexual partners. A study conducted in Humera town showed that 13.7% of respondents were involved in risky sexual behaviors [15]. The higher level of risky sexual behavior was seen in studies conducted in Jimma zone (42.1%) [12]. A study conducted among Tiss Abay female students also showed a high (70.3%) level of risky sexual behavior when compared with current study [9]. Possible explanation could be attributed to the reasons mentioned above.

The current study also found three variables which were found to have association with risky sexual behavior at *p* value less than 0.05. These variables include respondents between the ages of 20 and 23, drinking alcohol, and having poor knowledge towards HIV/AIDS. Respondents who were in the age group of 20 to 23 were found to be five times more likely to engage in risky sexual behavior when compared with youth in the age group of 14 to 19 years old. This could be because of the physical and psychological maturity of older youths, and the risk taking behavior among these age groups, resulting in increased risk of involving in sexual practice as the age increases. Similar findings were also observed in a study conducted in Jimma University [22].

Youths who drink alcohol were more than two times more likely to engage in risky sexual behavior. This could be because youths who abuse substances like alcohol may have poor judgment, which may result in risky sexual behavior. Similar findings were also observed in studies conducted in Tiss Abay, southwest Ethiopia and northwest Ethiopia [9, 26, 27]. In addition, studies have showed that youths who drink alcohol are more likely to engage in risky sexual behaviors like having multiple sexual partners, performing unprotected sexual intercourse, and having sex with high risk partners like commercial sex workers [2, 16, 27–30].

Students who have poor knowledge towards HIV/AIDS were more than four times more likely to engage in risky sexual behavior. This could be because of the lack of knowledge on HIV transmission and on ways of HIV prevention and the cumulative risk to engage in unprotected sex.

This study has certain limitations. Due to the nature of the cross-sectional study design, it may be difficult to make a causal inference. There may be recall bias among respondents when responding to some of the variables in this study.

## 5. Conclusion and Recommendation

A large number of in-school youths were involved in risky sexual behaviors like early sexual initiation, having multiple sexual partners, inconsistence use of condom, and having sex with high risk partner (CSWs). These behaviors are predisposing youths to several forms of sexual and reproductive health problems. Factors which were found to have association with risky sexual behavior include age of respondent's (20 to 23 years old), alcohol drinking, and having poor knowledge towards HIV/AIDS. So, school and community based programs to strengthen preventive measures, like reducing substance abuse among youths and increasing their knowledge towards HIV/AIDS, are important. Youths should also be encouraged to attend the sexual and reproductive health centers, and such centers should be strengthened. Awareness creation on condom use and risk sexual behavior and its consequences should be done through the available media of communication and comprehensive education on sexual and reproductive health issues should be included to the existing curriculum for high school and preparatory students.

## Figures and Tables

**Table 1:** Sociodemographic characteristics of respondents among Jiga high school and preparatory school students, 2014.

Variables	Frequency	Percentage
*Sex*		
Male	135	45
Female	165	55

*Age*		
<18 years old	113	37.7
≥18 years old	187	62.3
Mean ± SD: 17.75 ± 1.37		

*Marital status*		
Ever married	24	8
Never married	276	92

*Religion*		
Orthodox	293	97.7
Muslim	7	2.3

*Ethnicity*		
Amhara	300	100

*Educational level*		
Grade 9	120	40
Grade 10	97	32.3
Grade 11	43	14.3
Grade 12	40	13.3

*Current living condition*		
With both mother and father	188	62.7
With father only or mother only	51	17
With relatives or friends	36	12
Alone	25	8.3

*Previous residence*		
Urban	186	62
Rural	114	38

*Father's educational status*		
Cannot read and write	81	27
Can read and write	160	53.3
Primary education (grades 1–8)	46	15.3
Secondary education (grades 9–12)	9	3
College level and above	4	1.3

*Mother's educational status*		
Cannot read and write	147	49
Can read and write	106	35.3
Primary education (grade 1–8)	34	11.3
Secondary education (grade 9–12)	10	3.3
College level and above	3	1

**Table 2:** Sexual history of respondents among Jiga high school and preparatory school students, 2014.

Variables	Frequency	Percentage
Ever had sex		
Yes	48	16
No	252	84

Reason to start sex		
Personal desire	13	27.1
Peer pressure	17	35.4
Influence of alcohol	2	4.2
Coercion	5	10.4
Economic problem	2	4.2
Do not know or do not remember	9	18.8

Age at first sexual intercourse		
<18 years old	27	56.3
≥18 years old	21	43.8
Mean ± SD = 17.2 ± 1.35		

Use condom at first sex		
Yes	8	16.7
No	40	83.3

Had sex with CSW		
Yes	3	6.3
No	45	93.8

Total number of sexual partners		
One	34	70.8
≥two	14	29.2

Risky sexual behavior		
No	256	85.3
Yes	44	14.7

Had sexual intercourse in the past 12 months		
Yes	28	58.3
No	20	41.7

**Table 3:** Knowledge on HIV/AIDS and condom use among Jiga high school and preparatory school students, 2014.

Variables	Frequency	Percentage
Ever heard of HIV/AIDS		
Yes	280	93.3
No	20	6.7

Modes of HIV transmission^*∗*^		
Sexual contact	264	88
Blood transfusion	206	68.7
Mother to child transmission	213	71
Injection with contaminated needles	216	72
Through contaminated instruments	190	63.3
Hand shaking	8	2.7
By air	4	1.3
Do not know	9	3

Healthy looking person can be infected by HIV		
Yes	211	70.9
No	54	18

Healthy looking HIV infected individual can transmit HIV to another person		
Yes	200	66.7
No	53	17.7
Do not know	47	15.7

Knowledge index		
Poor knowledge	108	36
Good knowledge	192	64
Mean ± SD: 8.3 ± 1.96		

Condoms prevent pregnancy		
Agree	193	64.3
Disagree	52	17.3
Do not know	55	18.3

Condoms make sex less enjoyable		
Agree	95	31.7
Disagree	43	14.3
Do not know	162	54

Condoms are easy to use		
Agree	77	25.7
Disagree	66	22
Do not know	157	52.3

Condom use is against my religion		
Agree	108	36
Disagree	78	26
Do not know	114	38

The price of condom is too high to use regularly		
Agree	42	14
Disagree	117	39
Do not know	141	47

Condoms prevent STIs if used properly		
Agree	141	47
Disagree	57	19
Do not know	102	34

^*∗*^Response does not add up to 100% due to multiple responses.

**Table 4:** Non exual risk behaviors among Jiga high school and preparatory school students, 2014.

Variables	Frequency	Percentage
Ever drunk alcohol		
No	168	56
Yes	132	44

Ever chew chat		
No	286	95.3
Yes	14	4.7

Ever smoke cigarette		
No	293	97.7
Yes	7	2.3

Ever use shisha/hashish		
No	295	98.3
Yes	5	1.7

**Table 5:** Factors associated with risky sexual behaviors among Jiga high school and preparatory school students, 2014.

Variables	Risky sexual behavior		COR (95% CI)	AOR (95% CI)	*p*-value
	No *n* (%)	Yes *n* (%)			
*Sex*					
Male	113 (83.7)	22 (16.3)	1	1	
Female	143 (86.7)	22 (13.3)	0.79 (0.42, 1.49)	0.49 (0.16, 1.52)	0.213

*Age*					
14–19 years old	233 (87.6)	33 (12.4)	1	1	
20–23 years old	23 (67.6)	11 (32.4)	**3.4 (1.51, 7.56)**	**5 (1.59, 15.98)**	**0.006**

*Educational level*					
Grade 9	99 (82.5)	21 (17.5)	1	1	
Grade 10	89 (91.8)	8 (8.2)	0.64 (0.27, 1.49)	0.44 (0.17, 1.14)	0.090
Grade 11	38 (88.4)	5 (11.6)	**0.27 (0.09, 0.75)**	0.91 (0.26, 3.13)	0.879
Grade 12	30 (75)	10 (25)	0.39 (0.12, 1.28)	0.63 (0.19, 2.09)	0448

*Previous residence*					
Urban	155 (83.3)	31 (16.7)	1.55 (0.78, 3.11)	1.36 (0.57, 3.21)	0.488
Rural	101 (88.6)	13 (11.4)	1		

*Father's educational status*					
Cannot read and write	204 (84.6)	37 (15.4)	0.99 (0.21, 4.69)	1.73 (0.18, 16.51)	0.634
Primary education (grades 1–8)	41 (89.1)	5 (10.9)	0.67 (0.11, 3.94)	0.59 (0.05, 6.49)	0.667
Secondary (grades 9–12) and college level education	11 (84.6)	2 (15.4)	1		

*Mother's educational status*					
Cannot read and write	216 (85.4)	37 (14.6)	0.94 (0.2, 4.42)	0.74 (0.079, 6.96)	0.794
Primary education (grades 1–8)	29 (85.3)	5 (14.7)	0.95 (0.16, 5.63)	1.009 (0.097, 10.53)	0.994
Secondary (grades 9–12) and college level education	11 (84.6)	2 (15.4)	1		

*Ever drunk alcohol*					
No	153 (91.1)	15 (8.9)	1		
Yes	103 (78)	29 (22)	**2.87 (1.47, 5.62)**	**2.48 (1.13, 5.41)**	**0.023**

*Ever chew chat *					
No	249 (87.1)	37 (12.9)	1		
Yes	7 (50)	7 (50)	**6.73 (2.23, 20.28)**	2.76 (0.69, 11.09)	0.152

*Ever use shisha/hashish*					
No	254 (86.1)	41 (13.9)	1		
Yes	2 (40)	3 (60)	**9.29 (1.51, 57.32)**	3.33 (0.35, 31.95)	0.297

*Knowledge towards HIV/AIDS*					
Poor knowledge	80 (74.1)	28 (25.9)	**3.85 (1.97, 7.51)**	**4.53 (2.06, 9.94)**	**0.000**
Good knowledge	176 (91.7)	16 (8.3)	1	1	

*Living arrangement*					
Living with parents	200 (83.7)	39 (16.3)	1	1	
Living away from parent	56 (91.8)	5 (8.2)	0.46 (0.17, 1.22)	0.49 (0.16, 1.52)	0.213
